# Supplementary material for: Inactivation of the UL37 Deamidase Enhances Virus Replication and Spread of the HSV-1(VC2) Oncolytic Vaccine Strain and Secretion of GM-CSF
Source: Viruses. 2023 Jan 27;15(2):367. doi: 10.3390/v15020367 (PMC9961126; doi:10.3390/v15020367)
Supplement: Supplementary file 1 [file viruses-15-00367-s001.zip › viruses-2145178-supplementary.pdf]

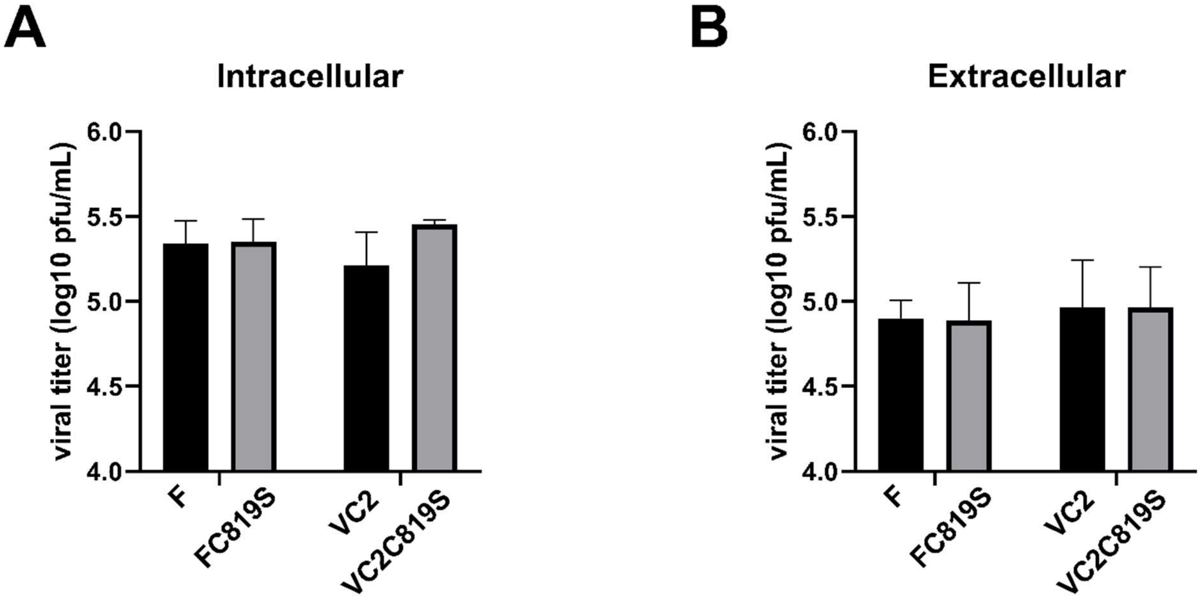

Figure S1. Intracellular and extracellular virion production. Cells were infected at an MOI of 1.0 and incubated for 24 hours post-infection. Infected cell media (A) and infected cell lysates (B) were collected and titrated on Vero cells. Error bars represent the standard error of the mean based on three experimental replicates.

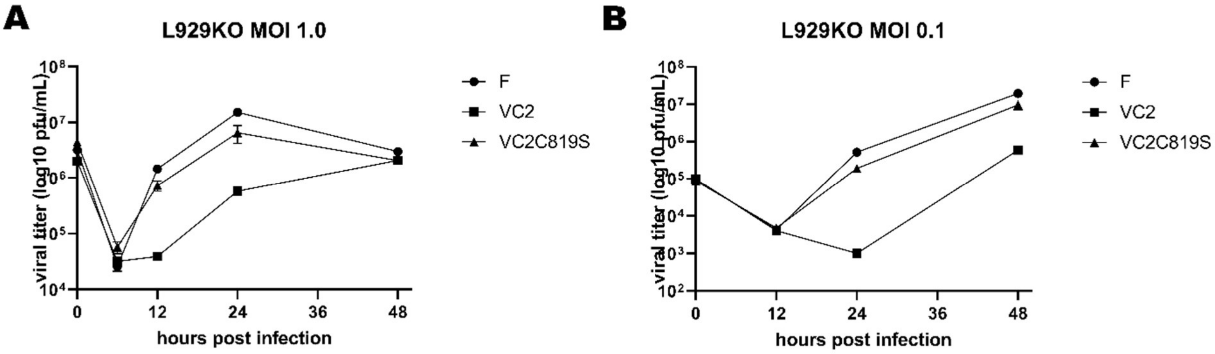

Figure S2. Replication kinetics of wild-type and recombinant viruses in L929KO cells. Growth curve of F, VC2, and VC2C819S at an MOI of 0.1 (A) and 1.0 (B) on L929 cGAS-/- cells. Whole lysates were collected at 0, 6, 12, 24, and 48 hpi and titrated on Vero.

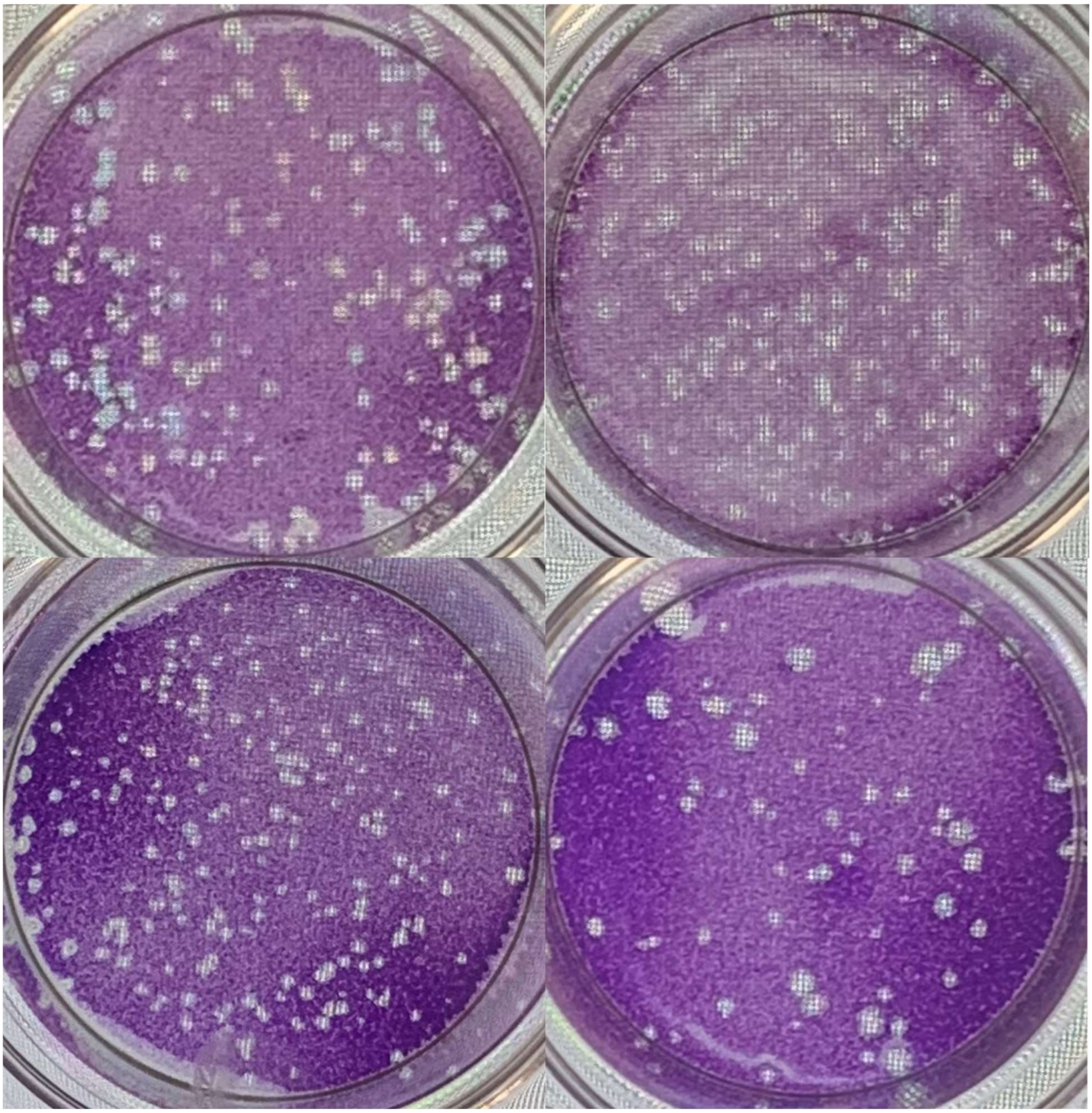

Figure S3. Plaque morphology on Vero cells. F (A), FC819S (B), VC2 (C), and VC2C819S (D) were serial diluted into a monolayer of Vero cells in a 12 well plate for 72hpi and then stained with 1% crystal violet.
